# Supplementary material for: Banana Peel Powder Biosorbent for Removal of Hazardous Organic Pollutants from Wastewater
Source: Toxics. 2023 Aug 1;11(8):664. doi: 10.3390/toxics11080664 (PMC10459949; doi:10.3390/toxics11080664)
Supplement: Supplementary file 1 [file toxics-11-00664-s001.zip › toxics-2509845-supplementary.pdf]

Supplementary material

Banana peel powder biosorbent for removal of hazardous organic pollutants from wastewater

Kelly C. S. Farias[a], Rita C. A. Guimarães [b], Karla R. W. Oliveira[c], Carlos E. D. Nazário[c], Julio A. P. Ferencz[a,d] and Heberton Wender[a*]

[a]Nano&Photon Research Group, Laboratory of Nanomaterials and Applied Nanotechnology (LNNA), Institute of Physics, Federal University of Mato Grosso do Sul, Campo Grande, Mato Grosso do Sul, 79070-900, Brazil
[b]Graduate Program in Health and Development in the Midwest Region, Medical School, Federal University of Mato Grosso do Sul, Campo Grande 79070-900, Brazil
[c]Institute of Chemistry, Federal University of Mato Grosso do Sul, 79070-900, Campo Grande, MS, Brazil
[d]Faculty of Engineering, Architecture and Urbanism and Geography, Federal University of Mato Grosso do Sul, 79070-900, Campo Grande, MS, Brazil.

*Corresponding authors: heberton.wender@ufms.br

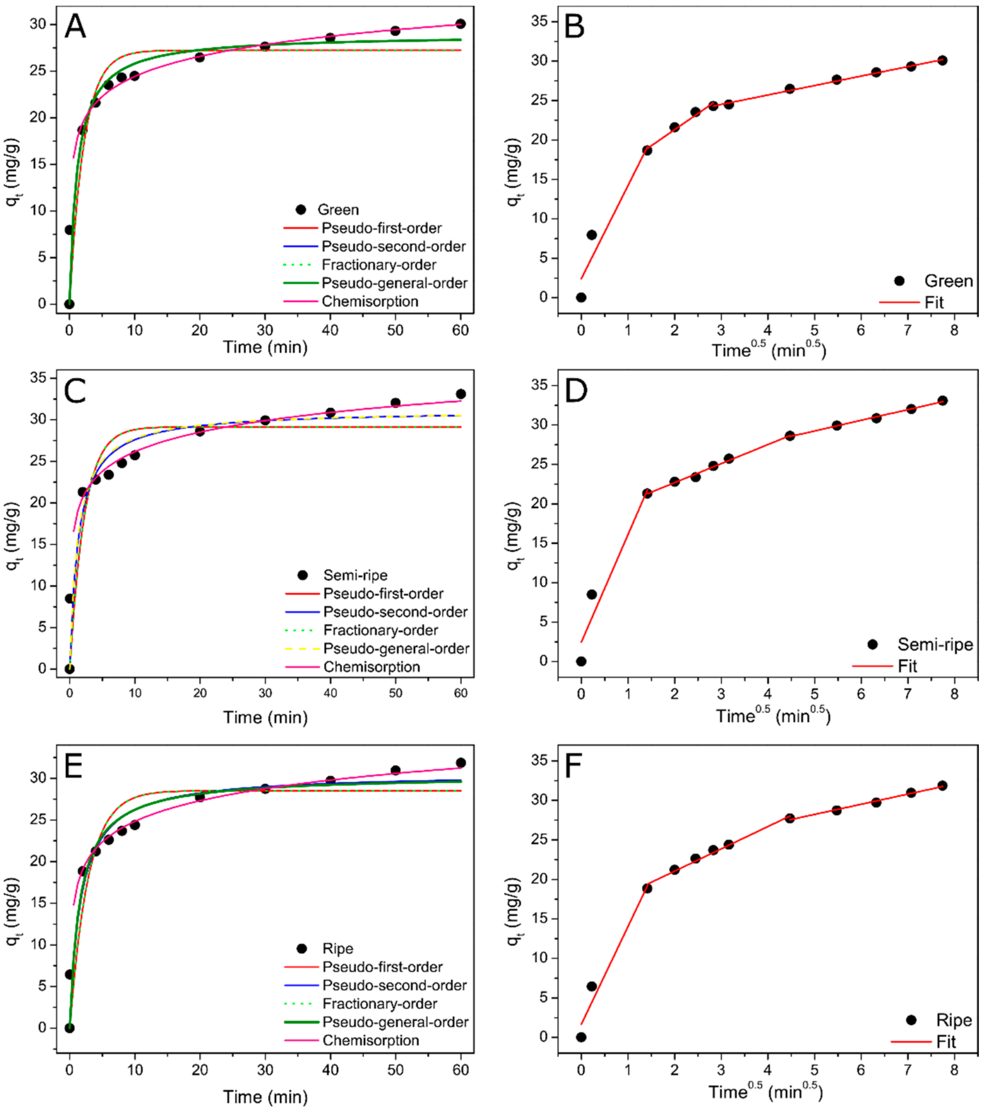

Figure S1. Kinetic models and intraparticle diffusion fits for MB removal using green (A, B), semi-ripe (C, D), and ripe (E, F) BPP. Conditions: 50 mg of BPP (biosorbent) and 50 mL MB dye solution at C_0 = 50 mg L^{-1}.

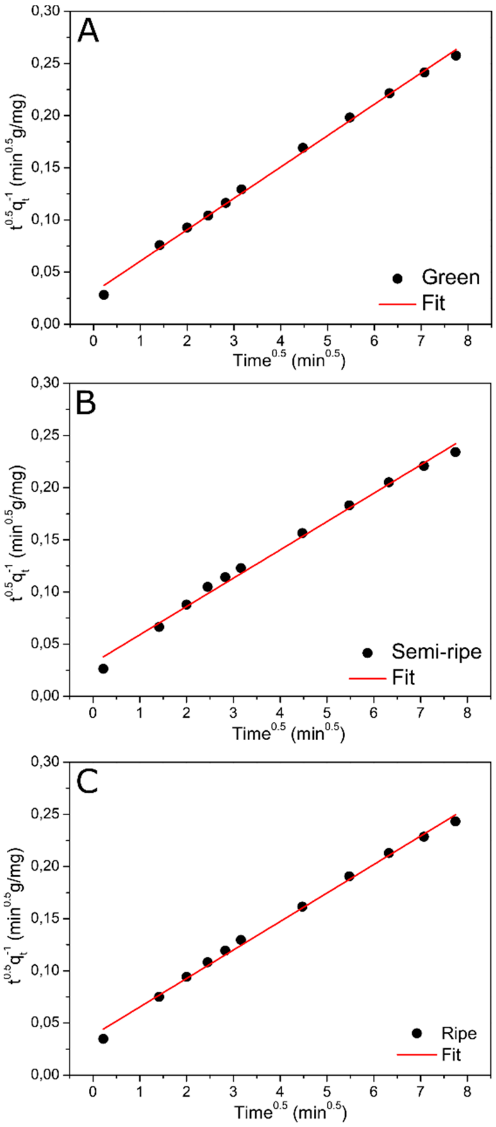

Figure S2. Fitted curves of the diffusion-chemisorption model for green (A), semi-ripe (B), and ripe (C) BPP. Conditions: 50 mg of BPP (biosorbent) and 50 mL MB dye solution at C_0 = 50 mg L^{-1}.

Table S1. Parameters obtained from the diffusion-chemisorption model for MB removal using green, semi-ripe, and ripe banana peel flour as biosorbent.

**Parameters**	**Ripeness stage**		
	Green	*Semi-ripe*	*Ripe*
**Diffusion-chemisorption**			
$k_{DC}(mg\ g^{-1}\ min^{-0.5})$	32.76 ± 2.78	31.40 ± 3.58	26.25 ± 1.98
$q_e(mg\ g^{-1})$	33.26 ± 0.63	36.85 ± 1.08	36.61 ± 0.84
R^2_{adj}	0.9965	0.9916	0.9948
Residual sum of squares	$1.92 \cdot 10^{-5}$	$3.79 \cdot 10^{-5}$	$2.37 \cdot 10^{-5}$

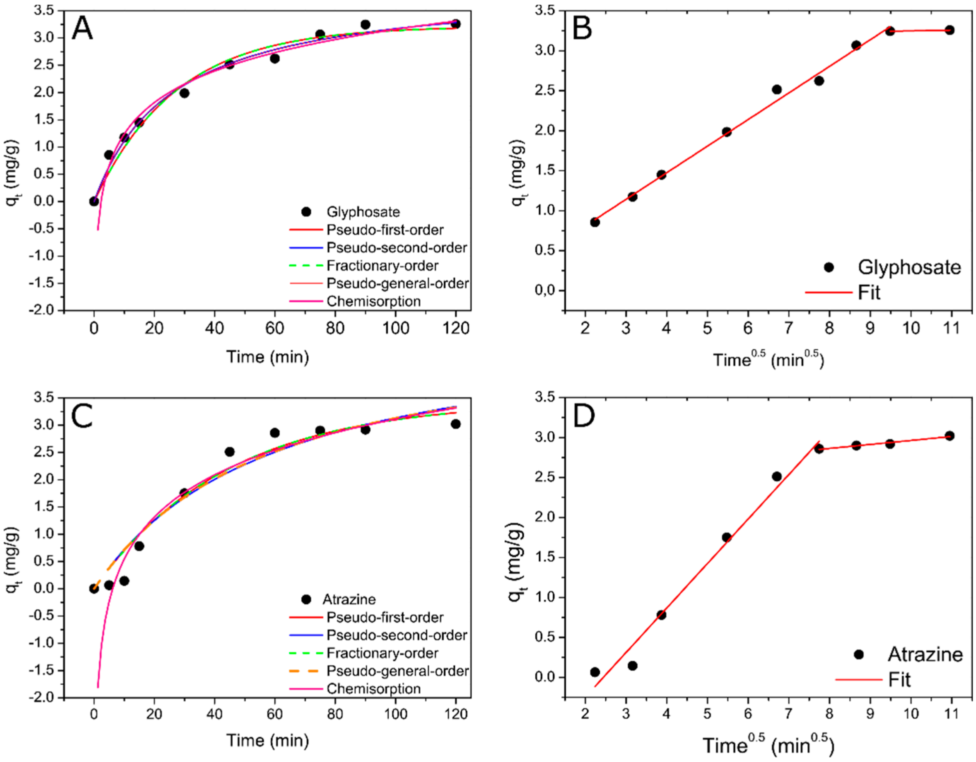

Figure S3. Fitted curves of the different kinetic models studied for semi-ripe BPP using glyphosate (A) and atrazine (C) as a pollutant and the intraparticle diffusion fit for glyphosate (B) and atrazine (D). Conditions: 60 mg of semi-ripe BPP and 10 mL of the pesticide at C_0 = 20 mg L^{-1}.
